# 

**Published:** 1922-09

**Authors:** 


### HOSPITAL and HEALTH

REVIEW

*Established 1886* *No.* 1854. *Vol. LXXI.*

*New Series.* Vol. 1. No. 12 *September* 1922 Price Sixpence

CONTENTS.

| | PAGE |
|---|---|
| NOTES AND COMMENTS—The British Medical Association Climbs Down—Asylum Reform—Premature Sanatorium Discharges—A Self-supporting Tuberculosis Colony—The Danger of Gall-Stones—The Maternity Bonus in Australia—Refuse Disposal—A Statesman's Speech—The Sale of Unsound Food—The Smoke Abatement Bill—The Milk and Dairies Act—National Health Insurance Medical Benefit—The Edinburgh School of Social Study and Training—Health Education in the United States—Unions and Divisions in Asylum Staffs—Convalescents in General Hospitals—The Hospital Saving Association—Should Patients Supply their Own Bread ?—Payment of Hospital Medical Staffs—The Trouble with Opticians—Extensions at University College Hospital—Queen Mary's Maternity Home—A Discerning Founder—The Spirit of the Staff | 339 |
| THE PUBLIC HEALTH: INTERVIEWS WITH LOCAL AUTHORITIES. III.—THE CITY OF BIRMINGHAM | 346 |
| THE SURGERY QUEUE. By R. W. Harris. | 348 |
| OUR PENAL AND PRISON SYSTEM.—I. | 349 |
| "ON THE STATE OF PUBLIC HEALTH" | 352 |
| UNEMPLOYMENT AND HEALTH | 354 |
| THE HISTORY OF MEDICAL ATTENDANCE ON THE POOR | 355 |
| THE HOSPITAL MANAGEMENT AND TREATMENT OF INFECTIOUS DISEASES.—I. By A. E. Pearson, L.R.C.P., M.R.C.S. | 356 |
| THE VOLUNTARY HOSPITALS COMMISSION | 358 |
| HOSPITAL FINANCE | 361 |
| HOSPITAL NEWS | 363 |
| REVIEWS OF BOOKS | 364 |
| CORRESPONDENCE—Do the Approved Societies Seek to Control the Doctors ?—Salisbury and District Infirmary and Hospital League—Home Nursing for Insured Persons | 366 |
| PARLIAMENTARY QUESTIONS AND ANSWERS | 368 |
| RECENT APPOINTMENTS | 368 |
| THE MEDICAL CURRICULUM | 369 |
| COURSE OF STUDY FOR TRAINED HOSPITAL NURSES | 374 |

